# Pathogenesis, histopathologic findings and treatment modalities of lipoprotein glomerulopathy: A review

**DOI:** 10.1590/2175-8239-JBN-2018-0148

**Published:** 2018-11-08

**Authors:** Eduardo Cambruzzi, Karla Lais Pêgas

**Affiliations:** 1 Santa Casa de Misericórdia de Porto Alegre Departamento de Patologia Porto AlegreRS Brasil Santa Casa de Misericórdia de Porto Alegre, Departamento de Patologia, Porto Alegre, RS, Brasil.; 2 Hospital Nossa Senhora da Conceição Porto AlegreRS Brasil Hospital Nossa Senhora da Conceição, Porto Alegre, RS.; Universidade Luterana do Brasil CanoasRS Brasil Universidade Luterana do Brasil, Canoas, RS, Brasil.

**Keywords:** Renal Insufficiency, Chronic, Lipoprotein, Kidney Diseases, Apolipoprotein, Nephrotic Syndrome, Fenofibrate

## Abstract

Lipoprotein glomerulopathy (LPG) is an uncommon cause of nephrotic syndrome and/or kidney failure. At microscopy, LPG is characterized by the presence of lipoprotein thrombi in dilated glomerular capillaries due to different ApoE mutations. ApoE gene is located on chromosome 19q13.2, and can be identified in almost all serum lipoproteins. ApoE works as a protective factor in atherosclerosis due its interaction with receptor-mediated lipoprotein clearance and cholesterol receptor. Most common polymorphisms include ApoE2/2, ApoE3/2, ApoE3/3, ApoE4/2, ApoE4/3, and ApoE4/4. All age-groups can be affected by LPG, with a discrete male predominance. Compromised patients typically reveal dyslipidemia, type III hyperlipoproteinemia, and proteinuria. LPG treatment includes fenofibrate, antilipidemic drugs, steroids, LDL aphaeresis, plasma exchange, antiplatelet drugs, anticoagulants, urokinase, and renal transplantation. Recurrence in kidney graft suggests a pathogenic component(s) of extraglomerular humoral complex resulting from abnormal lipoprotein metabolism and presumably associated to ApoE.

## Introduction

Lipoprotein glomerulopathy (LPG) is a rare autosomal recessive disorder, which determines marked proteinuria and progression to kidney failure. The compromised glomeruli exhibit ectatic capillary lumina occupied by lipoprotein thrombi.^1^^,^^2^^,^^3^ LPG typically compromises Asian patients, in special Japanese, and males outnumber females two to one. Serum levels of lipoprotein are typically increased especially β-lipoprotein and pre-β-lipoprotein.^2^^,^^4^^,^^5^^,^^6^ LPG can resemble type III hyperlipoproteinemia, but atherosclerosis, corneal arcus, and cutaneous xanthomas are very uncommon. Apolipoprotein (ApoE) is a component of human lipoproteins, with a molecular weight around 39kD, which serves as a ligand for cellular uptake of triglyceride-rich lipoproteins through specific receptors of the LDL receptor.^7^^,^^8^^,^^9^^,^^10^ Identification of ApoE isoforms E2/3, E2/4, E3/3, and E4/4 can establish the diagnosis of LPG. Adults are primarily affected by LPG, especially males (2:1 to female).^1^^,^^4^^,^^5^^,^^7^^,^^11^^,^^12^

The first case of LPG described in English literature was by Saito et al, who reported a patient with resistant nephritic syndrome and accumulation of lipid droplets in glomeruli loops.^9^ Steroid-resistant nephrotic syndrome associated to severe proteinuria is the clinical hallmark of LPG. The disease progresses slowly to kidney failure in approximately 50% of the affected patients, and recurrence in renal allografts can also be found.^1^^,^^4^^,^^5^^,^^7^^,^^12^^,^^13^^,^^14^^,^^15^^,^^16^

### Pathogenesis

ApoE is a fundamental component of lipid and lipoprotein metabolism by functioning as the ligand for receptor-mediated catabolism of chylomicrons, some HDLs, and VLDLs. ApoE is present in almost all serum lipoproteins and acts as a protective factor in atherosclerosis due its interaction with receptor-mediated lipoprotein clearance and cholesterol receptor.^1^^,^^3^^,^^4^^,^^7^^,^^8^^,^^17^ ApoE gene is located on chromosome 19q13.2, and has three common alleles: e2, e3, and e4. The ApoE gene contains four exons and three introns, and its most common polymorphisms are ApoE2/2, ApoE3/2, ApoE3/3, ApoE4/2, ApoE4/3, and ApoE4/4. There are three main isoforms: E2, E3 and E4, which differ in one amino acid substitution. ApoE is a 34-kDa protein with 299 amino acids that mediates tissue uptake of lipoprotein through LDL receptor-related protein and LDL receptor.^3^^,^^4^^,^^5^^,^^7^^,^^12^^,^^15^^,^^18^^,^^19^ Around 90% of serum ApoE is synthetized by hepatocytes and 10% by macrophages. At microscopy, LPG is characterized by intra-glomerular lipoprotein thrombi and type III hyperlipoproteinemia due to heterozygote mutation of ApoE gene. Up to now, sixteen different mutations of ApoE gene have been identified in patients with LPG (eleven missense, four amino acid deletions, and one amino acid duplication). Most of these mutations are located in, or close to, the LDL receptor-binding domain.^1^^,^^4^^,^^5^^,^^9^^,^^19^^,^^21^^,^^22^^,^^23^^,^^24^ Among the missense mutations, four are proline, four are arginine substitutions (at position 145, 147, 150, and 158 of the mature protein), and three are cysteine for arginine substitutions (at position 25, 114, and 150 of the mature protein).^4^^,^^7^^,^^8^^,^^19^^,^^25^^,^^26^^,^^27^^,^^28^ Deletions involve the region encompassing the amino acid residues 141-146 (141-143, 142-144, and 144-146 in the central region of the binding domain) or the region encompassing the amino acid residues 156-173 (which includes the Arg172 residue involved in the binding to LDL receptor).^13^^,^^15^^,^^17^^,^^19^^,^^20^^,^^23^^,^^29^^,^^30^ One amino acid duplication that has been recently reported involves the residue Asp151.^31^

Arg25Cys is a common mutation of ApoE gene and is known as ApoE Kyoto.^12^^,^^19^^,^^21^ Hu et al. presented 35 LPG patients carrying the ApoE Kyoto allele in southwest China, making it a frequent mutation related to LPG.^21^ Hu performed a family study and found that the patient's mother was a heterozygous carrier of apoE Kyoto and his father was a carrier of Cys112Arg. The author proved that the mutated genes of this patient were inherited from both of his parents. Above all, his parents were healthy to date and had not shown any symptoms of diseases. ^([21])^ Matsunaga et al.^33^ and Rovin et al.^34^ showed similar findings. Rovin et al.^34^ established that the ApoE mutation appeared to be sufficient to lead to glomerular lipoprotein deposition but not to clinical LPG. Li et al. suggested that both Arg25Cys and Cys112Arg are pathogenic mutations, although they have no evidence to establish that these mutations independently or together contribute to the pathogenesis of LPG. It is possible that there was a dose effect on apoE mutation induced by LPG. That is, co-occurrence of two mutations (two chromosomes respectively carrying a mutation) induces the relatively obvious clinical manifestations.^35^

Chen at al.^36^ examined the 5.5 kb genomic DNA encompassing the entire ApoE locus and adjoining flanking regions in 17 Chinese LPG patients and concluded that there was no ApoE gene mutation in these LPG patients. Therefore, ApoE gene mutation might not be the only cause of LPG. The finding of 64 proband family members determined as mutation carriers but that did not develop LPG, and the significant difference in lipoprotein profile between people with or without LPG and carrying the same ApoE mutations favor the hypothesis that the presence of an abnormal ApoE is necessary but is not the only determinant in the development of LPG.^36^ The factors possibly related to a more pronounced lipoprotein remnant accumulation and clinical expression of the disease can be: a) additional allelic variants in exons, introns, or regulatory regions of ApoE (located in *cis* or in *trans*) that can induce different levels of expression of the mutant vs the wild-type allele (a higher expression of the mutant ApoE allele determines a higher plasma level of ApoE-containing lipoproteins, which can aggregate in glomeruli); b) an uncommon mutation or a polymorphism in another gene possibly associated to the full phenotypic expression of LPG; and c) epigenetic processes related to the regulation of a mutant gene.^31^ In an animal model, Kanamaru et al.^37^ found LPG-like glomerular lesions induced by the chronic graft-versus-host reaction in Fcγ receptor (FcRγ)-deficient mice. Furthermore, Ito et al.^38^ generated LPG-like changes in ApoE and FcRγ double-knockout mice by injecting various apoE vectors. These results suggest that macrophage impairment may be one of the mechanisms responsible for the development of lipoprotein thrombi and the absence of macrophages in LPG.^2^

The most common mutation is the Sendai form, which is characterized by a substitution of proline for arginine-145. ApoE Sendai can break the α-helical structure of ApoE in the low-density lipoprotein receptor-binding domain and modify the ApoE protein that are deposited in glomeruli thrombi and mesangium.^2^^,^^7^^,^^8^^,^^19^^,^^21^^,^^39^^,^^40^ In ApoE Kyoto, a substitution of cysteine for arginine-25 can be found.^19^^,^^21^^,^^34^^,^^41^^,^^42^ The isoforms E2 and E4 can also be implicated with atherosclerosis. E3 isoform protein is commonly found in the general population and is considered a "neutral" phenotype. E4 isoform is associated to an increased risk of Alzheimer's disease. ApoE2 displays less than 1% binding affinity for the hepatic LDL receptor.^3^^,^^8^^,^^13^^,^^14^^,^^15^^,^^17^^,^^21^^,^^28^^,^^38^ ApoE Kyoto facilitates lipoprotein deposition in glomerular capillaries due to increased endothelial cell binding.^19^^,^^21^^,^^34^^,^^41^^,^^42^

### Histopathologic findings

**Figure 1:**
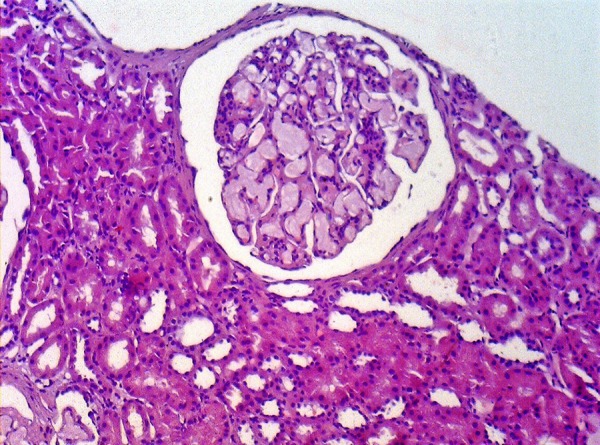
Lipoprotein glomerulopathy: A large glomeruli showing a pale eosinophilic material in the capillary lumina. Hematoxylin-eosin, 200x.

**Figure 2:**
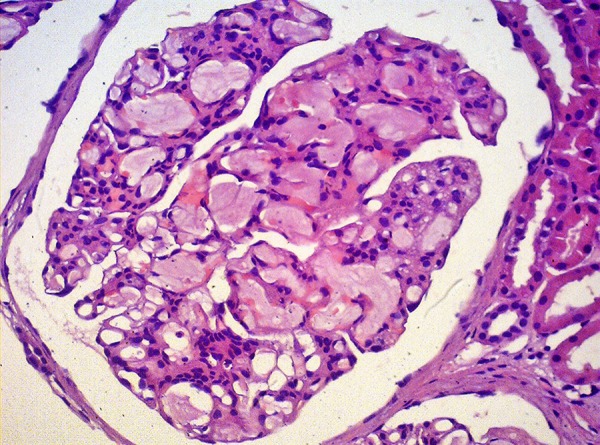
Lipoprotein glomerulopathy: Dilated capillary loops exhibiting an eosinophilic lipoprotein thrombi in the capillary lumina. Hematoxylin-eosin, 400x.

The characteristic histologic finding of LPG is the presence of large glomeruli due to ectatic capillary loops, which are occupied with lipoprotein thrombi (Figure 1). The glomeruli lesion is associated to polymorphisms and mutant isoforms of ApoE. Deficiency in intraglomerular lipoprotein uptake by mononuclear cells and disturbance in LDL receptor binding seems to be the possible mechanism involved with glomeruli damage.^1^^,^^5^^,^^6^^,^^9^^,^^16^ Macrophage activation and lipoprotein deposits are related to mesangiolysis. Typically, compromised glomeruli in LPG exhibits a pale eosinophilic lipoprotein thrombi in glomerular capillary loops, which are markedly dilated (Figure 2). Glomeruli thrombi are periodic acid/silver methenamine-positive and weakly periodic acid-Schiff positive, and must be differentiated with fibrin-thrombi and amyloid deposition. Oil red O or Sudan techniques demonstrates lipid droplets in glomeruli thrombi.^2^^,^^7^^,^^9^^,^^19^^,^^22^^,^^25^ Ultra-structural analysis shows that lipoprotein thrombi are concentrically lamellated, with small lipid vacuoles. Podocyte damage is related to proteinuria/nephrotic syndrome, and mesangial hipercellularity is associated to double contour. The mutations of ApoE more frequently implicated with LPG are ApoE Sendai (Arg145Pro), ApoE Kyoto (Arg25Cys), ApoE Tokyo, ApoE1, ApoE Guangzhou (Arg150Pro), ApoE Maebashi, ApoE Tsukuba, ApoE Chicago, and ApoE Okayama.^4^^,^^6^^,^^15^^,^^23^^,^^26^^,^^30^^,^^34^ Abnormal apoE proteins determine mesangial and basement membrane alterations, which are associated to increased glomerular permeability and nephrotic syndrome with higher levels of LDL, VLDL, and apolipoproteins B, C-II, and C-III. Mild glomerulomegalia, focal segmental sclerosis, mesangial proliferation, and focal reduplication of capillary basement membrane with mesangial interposition can be found in some cases. No macrophage foam cell can be identified in glomeruli/kidney interstitium.^1^^,^^7^^,^^10^^,^^11^^,^^15^^,^^16^^,^^22^^,^^28^^,^^43^ Positive immunoexpression for ApoB/ApoE antibodies is found in glomerular thrombi in occasional samples. No deposits of immunoglobulins or complement are identified in conventional immunofluorescence technique. IgA deposition can be identified in rare cases. The compromised kidney can show glomerulosclerosis and glomerular loss, which can determine chronic renal failure. Marked compromised kidneys can demonstrate interstitial and periglomerular fibrosis.^4^^,^^6^^,^^7^^,^^9^^,^^23^^,^^27^^,^^32^ Differential diagnosis include deficiency of lecithin-cholesterol acetyltransferase (which shows "bullous" capillaries, a vacuolted mesangium and foam cells in mesangium and capillaries), and fat emboli (round globules of fat in glomeruli loops, with scant or absence of apolipoprotein component, and without laminated appearance by electron microscopy).^1^^,^^9^^,^^11^^,^^15^^,^^17^^,^^32^^,^^44^

### Clinical findings

Associated to histological findings, proteinuria, dyslipidemia, and increased serum apolipoproteins levels are also hallmarks of LPG. The patients exhibit type III hyperlipoproteinemia and progress to nephritic syndrome in most cases. All age groups can be affected in LPG, with a discrete male predominance.^1^^,^^5^^,^^8^^,^^15^^,^^21^^,^^25^^,^^32^ Most patients in LPG are not affected by cutaneous xanthomas and atherosclerosis. Patients related to type III hyperlipidemia usually exhibit severe dyslipidemia, cutaneous xanthomas, prominent atherosclerosis, and ApoE homozygosity. Kidney involvement in type III hyperlipidemia is a very uncommon process characterized by mesangial and interstitial foam cell accumulation. Kidney biopsy is mandatory for the diagnosis of LPG, since there is no specific clinical or laboratory finding.^5^^,^^8^^,^^15^^,^^21^^,^^25^^,^^32^^,^^45^^,^^46^

### Treatment

Many reports describe various therapies such as LDL aphaeresis, plasma exchange, renal transplantation, steroids, antiplatelets, anticoagulants, ACEI, ARB, urokinase, and antilipidemic drugs.^4^^,^^8^^,^^18^^,^^21^^,^^32^^,^^35^^,^^39^ Leiri et al.^39^ and Arai et al.^40^ treated LPG patients with hyperlipidemia using intensive therapy with lipid-lowering agents. After treatment, both patients showed a remarkable decrease in urinary protein excretion, improvement in hyperlipidemia, and disappearance of the lipoprotein thrombi in the glomeruli by renal biopsy after 11 months to 2 years.^39^^,^^40^ The clinical effectiveness of fibrates was reported in two other patients.^41^^,^^42^ Matsunaga et al.^43^ initially treated a Japanese four-year-old female patient who had been in a nephrotic condition with hematuria, which was diagnosed as LPG based on pathological and molecular examination and treated with probucol, enalapril, and dipyridamole. This author found a decrease in the level of ApoE for a 1-year period. No improvement occurred in her nephrotic status. After, probucol was replaced with bezafibrate and atorvastatin calcium hydrate and valsartan were added. ApoE and total cholesterol decreased, and serum albumin increased over the subsequent 4-year treatment.^43^ Fibrates, agonists of the peroxisome proliferatoractivated receptor alpha (PPAR-alpha) receptor, decreased the level of HDL, remnant particles, very low-density lipoprotein (VLDL) cholesterol, and hepatic triglyceride secretion via activation of PPAR-alpha signaling. Although some authors report improvement with lipid-lowering agents, an effective standard treatment for LPG has not yet been established.^4^^,^^21^^,^^32^^,^^35^^,^^39^ Russi et al.^45^ described a 60-year-old Caucasian woman with LPG and Apolipoprotein E_MODENA_ mutation that was treated successfully with low-density lipoprotein-apheresis with the Heparin induced extracorporeal lipoprotein precipitation system. Heparin-induced extracorporeal low-density lipoprotein (LDL) precipitation (HELP) is a selective and careful apheresis procedure. Through the application of heparin and lowering the pH value, lipoproteins and fibrinogen are reduced by 50-60%. In addition, adhesion molecules (ICAM-1, VCAM-1, p-selectin), which play a key role in the development and progression of atherosclerosis are also markedly reduced.^44^ In patients refractory to conventional treatment, LDL-apheresis is a valid therapeutic tool to be associated with drugs to rapidly reduce the serum lipid values and improve renal function, thereby reducing the toxic effect.^45^ Hamatani et al.^47^ reported two patients with LPG, a daughter and a mother, who were successfully treated with niceritrol. Both patients carried ApoE Tokyo/Maebashi mutation. One of these patients was treated with several medications including pravastatin, ethyl icosapentate, enalapril, warfarin, and cyclophosphamide, all of which failed to reduce her proteinuria. The pravastatin was changed to 500 mg/day of niceritrol, which was subsequently increased to 750 mg/day. After the initiation of niceritrol treatment, her urinary protein-to-creatinine ratio decreased to around 1.0 g/gCr, and her serum creatinine level decreased to around 0.7 mg/dL. Of note, not all patients respond well to niceritrol. Saito et al.^48^ reported a patient in whom niceritrol failed to prevent the worsening of renal function. Niacin also decreases TG and LDL cholesterol. Although the precise mechanism is still unclear, it is postulated that niacin inhibits lipolysis of TG in adipose tissue, which in turn reduces TG synthesis in the liver. Reduced TG then decreases VLDL and therefore LDL cholesterol formation.^49^ Recently, combined therapy with statin and extended release niacin was reported to cause a significant regression in the intima-media thickness of the carotid artery and to prevent major cardiovascular events.^47^ Xin et al.^50^ demonstrated that immunoadsorption onto protein A, a selective removal of immunoglobulins from patients in an extracorporeal circuit, was associated with a significant response shown by reduced proteinuria, decreased ApoE and resolved intraglomerular thrombi in thirteen patients with LPG, and hypothesized that repeat immunoadsorption might also be effective in recurrent patients. They suggest that immunoadsorption is an acceptable alternative treatment option in patients with LPG.

Although all these treatments determine some benefits to LPG patients, not all patients respond the same way. A point to be further analyzed is the heterogeneity of the disease and the type of mutation involved. Most mutations, including ApoE Sendai, associated with LPG locate around the receptor-binding domain for LDL cholesterol and reduce the receptor-binding activity. Patients frequently have accompanying hyperlipidemia. Moreover, several patients with ApoE Kyoto or ApoE5 mutations, which locate far from receptor binding sites, are not complicated with hyperlipidemia. Those mutations indicate that ApoE mutants causing LPG do not damage glomeruli via hyperlipidemia but might injure glomeruli directly by forming aggregated deposits of lipoproteins that have high affinity or low clearance in glomeruli.^1^^,^^4^^,^^8^^,^^12^^,^^21^^,^^32^^,^^35^ In addition to ApoE mutations, other factors, including mesangial or endothelial dysfunction and macrophage or fragment crystalizable (Fc) receptor abnormality are etiologically attributed to LPG.^43^ Due to heterogeneity and rarity of the disease, establishing an effective treatment is difficult, in part because the best therapy for a patient in particular is determined, in most cases, after unsuccessful previous treatment. In some patients, the disease is in advanced chronic stage. LPG patients should be stratified based on mutation type and other associated factors, and long-term follow-up from many therapeutic strategies should be reported.^1^^,^^4^^,^^8^^,^^12^^,^^21^^,^^32^^,^^35^^,^^43^

Since half of the patients with LPG might eventually develop end-stage renal disease, kidney transplantation should be considered in these patients. However, the long-term outcome of kidney transplantation in patients with LPG remains uncertain. All of five kidney transplants reported in the literature had LPG relapse, which were confirmed by renal graft biopsy within 2 years after transplantation. It seems that LPG recurrence in a transplanted kidney is inevitable, which is also associated with poor prognosis.^12^^,^^21^^,^^32^ Cheung et al.^12^ report a patient who suffered from ESRD with coexisting LPG and fibrillary GN and received deceased kidney transplant. The 10-year follow-up did not reveal any clinical features of disease recurrence. Recurrence in the transplanted kidney suggestes a pathogenic role of extraglomerular humoral component(s) resulting from abnormal lipoprotein metabolism, presumably linked to ApoE.^51^
